# Medicines Reconciliation in the Emergency Department: Important Prescribing Discrepancies between the Shared Medication Record and Patients’ Actual Use of Medication

**DOI:** 10.3390/ph15020142

**Published:** 2022-01-26

**Authors:** Tanja Stenholdt Andersen, Mia Nimb Gemmer, Hayley Rose Constance Sejberg, Lillian Mørch Jørgensen, Thomas Kallemose, Ove Andersen, Esben Iversen, Morten Baltzer Houlind

**Affiliations:** 1 The Capital Region Pharmacy, 2730 Herlev, Denmark; tanja.stenholdt.andersen@regionh.dk (T.S.A.); mia.nimb.gemmer@regionh.dk (M.N.G.); hayley.rose.constance.sejberg@regionh.dk (H.R.C.S.); 2 Emergency Department, Copenhagen University Hospital Amager and Hvidovre, 2650 Hvidovre, Denmark; lillian.moerch.joergensen@regionh.dk (L.M.J.); ove.andersen@regionh.dk (O.A.); 3 Department of Clinical Research, Copenhagen University Hospital Amager and Hvidovre, 2650 Hvidovre, Denmark; thomas.kallemose@regionh.dk (T.K.); esben.iversen@regionh.dk (E.I.); 4 Department of Clinical Medicine, Faculty of Health and Medical Sciences, University of Copenhagen, 2200 Copenhagen, Denmark; 5 Department of Drug Design and Pharmacology, University of Copenhagen, 2100 Copenhagen, Denmark

**Keywords:** shared medication record, medication reconciliation, drug information service, hospital pharmacy service, electronic prescribing, electronic medical record, clinical pharmacist, emergency department

## Abstract

Medication reconciliation is crucial to prevent medication errors. In Denmark, primary and secondary care physicians can prescribe medication in the same electronic prescribing system known as the Shared Medication Record (SMR). However, the SMR is not always updated by physicians, which can lead to discrepancies between the SMR and patients’ actual use of medication. These discrepancies may compromise patient safety upon admission to the emergency department (ED). Here, we investigated (a) the occurrence of discrepancies, (b) factors associated with discrepancies, and (c) the percentage of patients accessible to a clinical pharmacist during pharmacy working hours. The study included all patients age ≥ 18 years who were admitted to the Hvidovre Hospital ED on three consecutive days in June 2020. The clinical pharmacists performed medicines reconciliation to identify prescribing discrepancies. In total, 100 patients (52% male; median age 66.5 years) were included. The patients had a median of 10 [IQR 7–13] medications listed in the SMR and a median of two [IQR 1–3.25] discrepancies. Factors associated with increased rate of prescribing discrepancies were age < 65 years, time since last update of the SMR ≥ 115 days, and patients’ self-dispensing their medications. Eighty-four percent of patients were available for medicines reconciliations during the normal working hours of the clinical pharmacist. In conclusion, we found that discrepancies between the SMR and patients’ actual medication use upon admission to the ED are frequent, and we identified several risk factors associated with the increased rate of discrepancies.

## 1. Introduction

Medicines reconciliation is an essential task for preventing medication errors in both primary and secondary care [1,2,3,4]. It ensures correct and updated information about patients’ medication, which is especially important when patients transfer between sectors. Medicines reconciliation requires a detailed medication history, which includes examination of all recently dispensed prescriptions combined with patient interviews [5].

In Denmark, hospitals and primary care physicians (e.g., general practitioners, ophthalmologists, private dermatologists, etc.) have access to the Shared Medication Record (SMR), which is a central electronic database containing information about all medications prescribed and dispensed at a community pharmacy within the past two years for residents and citizens of Denmark [6,7]. The SMR provides an overview of the current medication status for all patients and gives the patient’s healthcare team access to up-to-date prescribing information [6,7]. For example, the SMR indicates whether a patient has a dosette box from the community pharmacist or receives help with dispensing medication via home care, district nurses, or care assistants. Furthermore, sales records in the SMR for purchased medications can also indicate patient non-compliance. If a physician involved in the patient’s treatment notices any obvious medication errors, they are required to fix the errors and update the SMR [8]. Altogether, the SMR aims to prevent medication errors by increasing accessibility to patients’ current medication status [6,9].

Discrepancies between the SMR and patients’ actual use of medicines can result in improper prescribing or medication errors, either during hospitalization or after discharge [10,11]. This is particularly relevant in acute settings where patients often cannot speak for themselves about their medication history [12]. In such cases, the SMR is a valuable resource for clinicians and pharmacists—but only if it is accurate. Therefore, it is always important to discuss and confirm a patient’s current medication status directly with the patient or their caregiver [10,13]. Ideally, medicines reconciliation should be performed within the primary sector to keep the SMR up to date and improve its reliability during acute admissions.

It is important to note that the SMR categorizes the patient’s medications into orders and prescriptions. When a patient is admitted to the hospital, any active orders in the SMR are automatically transferred to the hospital’s local electronic prescribing system. This does not include active prescriptions that are no longer connected to an order. The admitting physician must review all active orders in the SMR and consider whether the patient should continue to receive these medications during hospitalization [14]. This becomes problematic if the general practitioner (GP) has not reviewed the patient’s orders. For example, the SMR could contain an old order for a medication without a stop date or without an active prescription, which might indicate that the medication is no longer in use. If these orders are not corrected, they can be transferred to the hospital’s prescribing system and ultimately lead to improper prescribing of a medication the patient does not need. Prior to discharge, the physician must again consider which orders should be continued after discharge. Each time a change is made in the SMR at discharge (e.g., new order/prescription, deprescription, or change in dose/frequency), the physician is required to indicate that the SMR has been updated [14].

Previous studies have shown that the SMR is not used as intended by physicians during medicines reconciliation [15,16,17], but it is unknown how often discrepancies occur in the emergency department (ED). Therefore, the purpose of this study is to investigate (a) the number and types of discrepancies, (b) the factors associated with discrepancies, and (c) the number of medicines reconciliations that could realistically be completed by a clinical pharmacist.

## 2. Results

### 2.1. Patient Characteristics

A total of 117 patients were admitted to the ED during the study period. Of these, 17 were excluded due to no active orders/prescriptions in the SMR (*n* = 11), patient isolation (*n* = 3), discharge against medical advice (*n* = 2), or death during admission (*n* = 1). Medicines reconciliation and a complete medication review was completed for 100 patients: 51 primary, 40 secondary, and nine retrospective. Patient characteristics for the final study population (*n* = 100) are shown in Table 1. Median age was 66.5 years, and 52% of patients were men. Patients used a median of six (IQR: 3–9) regular medications and two (IQR: 1–3) PRN medications. Fifty-five patients were referred by emergency services or an out-of-hours healthcare professional, 37 were referred by a GP or outpatient clinic, and eight were self-referrals.

### 2.2. Number of Prescribing Discrepancies

**Figure 1:**
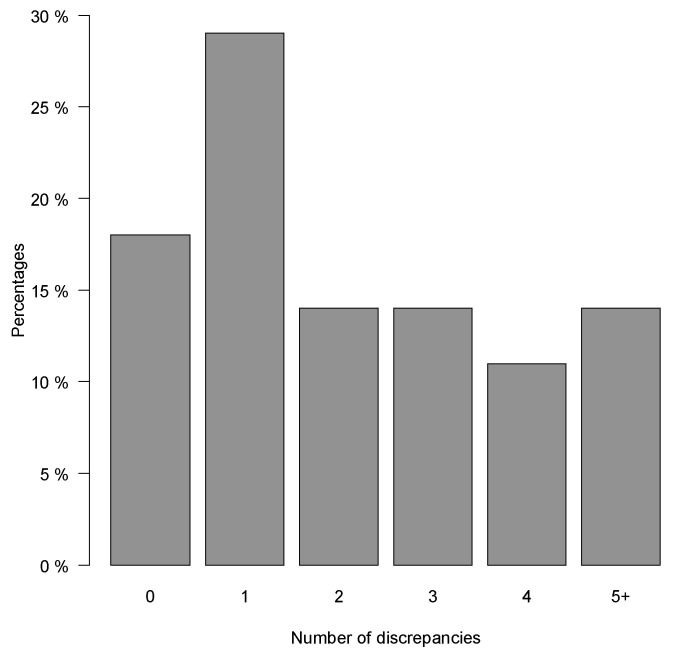
The percentage of patients with a specific number of discrepancies found between actual use of medication compared to the shared medication record (SMR).

From a total of 852 prescriptions (648 regular medications and 204 PRN medications), the clinical pharmacists identified 240 discrepancies between the SMR and patients’ actual use of medication during medicines reconciliation. Figure 1 shows the distribution of discrepancies per patient: 81% of patients had ≥1 discrepancy, while 14% had ≥5 discrepancies. The median number of discrepancies found per patients was two [IQR 1–3.25].

### 2.3. Types of Prescribing Discrepancies

Table 2 shows the most frequent types of discrepancies. The most common discrepancies were order no longer in use (65%), dosing frequency incorrect (15%), and order missing (12%). All discrepancies classified by anatomical therapeutic index (ATC) are shown in (Table A1). Discrepancies were most frequently observed for medications classified as A02 (antacids and certain laxatives) or N02 (analgesics such as opioids). Among the discrepancies involving opioids, four were orders no longer in use, and two were due to missing orders. Discrepancies involving medications classified as J01 (systemic antibiotics) included 14 cases where the indication for antibiotic treatment was no longer relevant.

### 2.4. Factors Associated with the Rate of Prescribing Discrepancies

Table 3 shows factors associated with the rate of discrepancies. Patients aged 65–80 and >80 both had reduced rates of discrepancies per medication listed in the SMR, 42% (CI: 29–52) and 51% (CI: 38–62), respectively, compared with patients aged < 65 years. Adjusting for age and sex, patients with ≥115 days since the previous SMR update had a 53% (CI: 29–82) higher discrepancy rate per medication listed in the SMR compared with patients with ≤27 days since the previous SMR update. Patients who required assistance with medication dispensing also had a 72% (CI: 65–78) reduced rate of discrepancies per medication listed in the SMR compared with patients who dispensed medication themselves. Patients who required assistance dispensing their medications also had a 72% (CI: 65–78) reduced discrepancy rate per medication listed in the SMR compared with patients who dispensed medication themselves. The prescribing discrepancy rate was not associated with the type of physician who last updated the SMR, the time of admission to the ED, or triage level. Sensitivity analysis excluding discrepancies due to order not in use only showed additional association for patients admitted outside of normal working hours, with a 159% (CI: 84–263) increased rate of discrepancies per medication listed in the SMR compared to patients admitted during normal working hours (Table A2).

### 2.5. Medicines Reconciliations Completed during Normal Working Hours

Time of admission and discharge from the ED are shown in Table 4. Forty-nine patients (49%) were admitted during normal working hours (8.00 a.m.–3.00 p.m.), and 51 patients (51%) were admitted outside of normal working hours. Among patients admitted outside normal working hours, 35 patients were still in the ED the following morning.

Therefore, it was possible for the clinical pharmacists to complete medicines reconciliation for 84 patients (84%) during normal working hours. Of these, 64 patients (76%) had ≥1 discrepancy found during medicines reconciliation.

## 3. Discussion

### 3.1. Main Findings

This study investigated the number and types of discrepancies found between the SMR and patients actual medication use upon acute admission to the ED. Clinical pharmacists identified a total of 240 prescribing discrepancies among 100 patients. The median number of discrepancies per patient was two, and 81% of patients had ≥1 discrepancy. The most common types of discrepancy found were order no longer in use, dosing frequency incorrect, and order missing. Factors associated that increased discrepancy rates included age < 65, and extended time since prior SMR update. The study also evaluated the percentage of medicines reconciliations that could be completed by a clinical pharmacist within working hours. Medicines reconciliation was possible for 84% of patients.

### 3.2. Results in Context of Other Studies

The frequency of discrepancies upon ED admission were lower than what has been observed in other Danish studies [15,16,18]. This may be explained by differences in clinical setting and inclusion criteria. For example, Buck et al. and Bülow et al. studied patients in the ED, geriatric ward, and the orthopedic surgery ward. Their inclusion criteria were age >50 years with ≥5 medications [15,18]. It has previously been demonstrated that increased medication use is associated with an increased risk of discrepancies [19,20,21,22], so the findings by Bülow et al. 2019 [16], Bülow et al. 2021 [15] and Buck et al. [18] may be related to the higher prevalence of polypharmacy. These studies did not find an association between age and the frequency of discrepancies found, which is likely due to the difference in clinical settings compared to our study. The study by Pippins et al. found that age <85 was associated with a higher risk of unintended medication discrepancies with potential for causing harm [23]. We found in our study that age <65 was associated with a higher frequency of discrepancies. However, in contrast to Pippins et al., we found that patients who required assistance with medication dispensing had a reduced rate of discrepancy compared to patients that dispensed medication themselves. This difference in findings between Pippins et al. and our study could be because of the Danish SMR system, where a similar tool was lacking in the Pippins et al. study. In Denmark, patients who receive help with dispensing their medication via home care, district nurses, or care assistants, get their medicines dispensed directly from orders in the SMR. The association between discrepancies and time since prior SMR update observed in our study is similar to findings from Bülow et al. 2021 [15]. Cornich et al. did not find a significantly higher discrepancy rate for admissions that took place outside of normal working hours [24]. Our study possibly indicates an increased discrepancy rate for patients admitted outside of normal working hours.

The types of discrepancies found in our study are comparable to other Danish studies [15,16,18,25]. We found that 65% of discrepancies were due to medication being no longer in use, which is similar to results from Bülow et al. 2019 [16] and Bülow et al. 2021 [15] but higher than results from Buck et al. [18]. We found that 15% of discrepancies were due to errors in dosing frequency. The two studies by Bülow [15,16] divide this category into two subcategories: PRN administration of regularly scheduled prescriptions, and regular scheduled administration of a PRN prescription. If these categories are merged, then the combined frequency of discrepancies found due to errors in dosing frequency from Bülow et al. 2019 [16] is similar to our study, but the frequency in Bülow et al. 2021 [15] is more than double what we observed. We found that 12% of discrepancies were due to an omission of order, which is similar to Bülow et al. 2019 [16] and Bülow et al. 2021 [15], but lower than what has been reported by Buck et al. [18] and Houlind et al. [25]. However, these studies use different terminology to describe the types of discrepancies, which makes direct comparison difficult. Finally, we found that antacids and analgesics were medication groups most frequently associated with prescribing discrepancies, which corresponds with the findings from Bülow et al. 2021 [15].

### 3.3. Updating the SMR: Possible Solutions and Reflections

The SMR can help healthcare professionals obtain an overview of a patient’s medication use, detect noncompliance, and help prevent medication errors. However, our results indicate that dosing discrepancies are common regardless of how a patient is referred to the ED. This suggests that relying solely on the SMR for a patient’s medication history is unsafe, which is supported by several other studies [26,27,28,29]. A valid medication history should include at least two information sources with different perspectives (i.e., prescribing and dispensing) [5,13]. Clinical pharmacists are essential for this purpose, as they have an opportunity to actively discuss medication use with the patient [2]. In addition, allowing clinical pharmacists to perform medicines reconciliation would enable physicians to focus on other aspects of patient care.

We found that 84% of patients were physically available for medicines reconciliation during normal working hours, meaning that medicines reconciliation combined with a medication review could in theory be performed for as many as 28 patients per day. However, previous studies have shown that medicines reconciliation takes approximately 30 min per patient (Buck et al.: 29 min, Urban et al.: 35.4 min, Cornich et al.: 24 min) [18,22,24], and a complete medication review would require even more time [30]. This suggests that a single person could perform no more than 14 medicines reconciliations per day. If the goal is to identify all discrepancies for all patients, then more staff resources must be dedicated. Alternatively, factors associated with prescribing discrepancies can be used to identify patients at highest risk for serious medication errors.

Accurate medicines reconciliation during admission increases the chances that the medication list will be updated at discharge. All physicians are expected to update the SMR any time they change a patient’s medication [8], but this does not always occur. Despite best practice guidelines, primary care physicians are not legally required to update the SMR [8]. In secondary care, updating the SMR is required by regional standard operating procedures [14]. In practice, maintaining an accurate electronic medication list is time consuming [31,32], and correct use of the SMR is limited by factors such as motivation, technical problems, time constraints, and familiarity with the electronic system [17].

Since patients potentially interact with many physicians across healthcare sectors, it must be made clear who has this responsibility for ensuring that the patient’s medication list is kept up to date [33]. Rose et al. suggest that a patient’s GP should be responsible for ensuring the SMR is kept up to date [31]. Unfortunately, no national agreement has been made within the primary sector in Denmark. Another solution could be to utilize clinical pharmacists, either in the hospital or in outpatient clinics. Hospital-based pharmacists could update the SMR at discharge, thereby preventing inappropriate prescriptions from continuing until the patient sees their primary care physician. Dedicated pharmacists in primary care could assist with medicines reconciliation for patients who are in a stable phase of their treatment, thereby preventing medication errors during future hospitalization. This pharmacist-based concept is utilized in other countries but remains uncommon in Denmark, in part because pharmacists in Denmark are not considered authorized healthcare professionals and, therefore, have limited access to the SMR. A third solution could be to promote patient involvement. For example, patients could be prompted on a yearly basis to review their own medication list to identify any prescriptions no longer in use. Increased patient involvement in general may also encourage dialogue between the patient and their GP that could help resolve any issues regarding medication compliance or inappropriate use.

### 3.4. Strengths and Limitations

The main strength of this study is that it identifies a daily clinical challenge in the ED regarding discrepancies found between the SMR and patients’ actual medication use. Furthermore, the study included patients on three consecutive days. This study also has some important limitations. First, the study was not designed to investigate the clinical significance or long-term consequences of prescribing discrepancies. Second, this was a single-center study and results are not necessarily generalizable to other healthcare settings. Third, we did not investigate how many discrepancies continued from admission to discharge, so we could not evaluate the effectiveness of a pharmacist-based intervention. The timing and duration of the study could also be considered a limitation, as there may be variation in the frequency of discrepancies found on different days of the week. Finally, our results rely on the accuracy of patients’ reported use of medication.

## 4. Materials and Methods

### 4.1. Ethics Approval

Data collection was performed during standard patient care as part of a quality improvement project by MNG, TSO and HRCS. Quality improvement projects in Denmark do not require prior ethical approval. The study was approved by a local committee at Copenhagen University Hospital, Amager and Hvidovre (WZ20017637-2020-77). All data were stored in anonymized form.

### 4.2. Setting

The tax-funded Danish healthcare system provides free and equal healthcare to citizens and residents of Denmark. Copenhagen University Hospital Amager & Hvidovre, Hvidovre, Denmark (hereafter Hvidovre Hospital) covers 10 municipalities with a population of approximately 550,000. Each year, the hospital has approximately 16,500 medical admissions, of which 85% are acute admissions to the ED. The Hvidovre Hospital ED is always open and has an acute medical ward with a capacity of 29 beds. Patients are typically referred to the acute medical ward by their GP, outpatient clinic, medical helplines, on-call/out-of-hours services, or by calling the emergency services. Patients can also be referred to the acute medical unit internally from other ED units. Patients can stay in the ED for up to three days before they are discharged or transferred to a specialized medical ward in the hospital.

The ED has permanent affiliations with pharmacy technicians, clinical pharmacists, physiotherapists, and doctors from a variety of medical specialties. During weekdays, pharmacy technicians dispense and administer medications and prepare discharge prescriptions between 7 a.m. and 2 p.m. Pharmacy technicians are often the first to notice specific medication issues, which are then referred to a clinical pharmacist. There is typically only one clinical pharmacist available between 8 a.m. and 3 p.m. The clinical pharmacist reviews and resolves any medication issues noted by the pharmacy technician. They also complete medicines reconciliation for as many patients as possible, prioritizing newly admitted geriatric patients and patients from particular medical specialties.

### 4.3. Design and Patients

The study included all patients age ≥18 years who were admitted to the Hvidovre Hospital ED on three consecutive days in June 2020. Exclusion criteria were: (i) no active orders/prescriptions in the SMR or no dispensed medication within the previous two years in the SMR, (ii) patient isolation, (iii) discharge against medical advice prior to interview with the clinical pharmacist, and (iv) death during admission.

### 4.4. Data Collection and the Best Possible Medication History

Three senior clinical pharmacists (≥5 years of experience) performed medicines reconciliation in the ED during the three-day period. For each patient, the clinical pharmacist recorded the patient’s sex, age, number of regular medications, and number of PRN medications. The SMR and electronic patient record were used to determine the time of admission, type of referral, triage level, time of discharge, and details about the most recent update of the SMR prior to admission. The clinical pharmacist then obtained a medication history for all prescribed and over-the-counter (OTC) medications as well as any vitamins and dietary supplements, noting whether the patient dispensed their own medication or received assistance. The medication history was collected from at least one prescribing source and one dispensing source. Prescribing sources included the SMR, dose dispensing card, or the patient’s GP. Dispensing sources included purchasing records from the SMR, patient interview, examination of medicine labels, or telephone contact with the patient’s relative, nursing facility, or district nurse. The purpose of locating the dispensing source was to identify any discrepancies between how a medication was prescribed and how it was used by the patient.

The medication history was categorized as primary, secondary, or retrospective: primary if the pharmacist completed medicines reconciliation before a physician transferred information from the SMR into the electronic prescribing system, secondary if the pharmacist completed medicines reconciliation after this transfer occurred, and retrospective if the pharmacist completed medicines reconciliation after patient discharge. Retrospective medication histories were obtained by contacting the patient by telephone.

### 4.5. Outcomes

The primary outcome was the number and types of discrepancies between the SMR and patients’ actual use of medication. A discrepancy was defined as any inconsistency between the SMR and the medication history obtained by the clinical pharmacist. Discrepancies were classified as: (a) order not in use, (b) incorrect dose frequency, (c) omission of order, (d) duplicate order, or (e) dosage incorrect. Discrepancies for vitamins and dietary supplements were only recorded if the SMR indicated they had been prescribed by a physician. Secondary outcomes were: (i) factors associated with discrepancies, and (ii) the percentage of patients available for medicines reconciliation by a clinical pharmacist during normal working hours.

### 4.6. Statistics

All patient characteristics are presented as medians with interquartile range (IQR) or frequency with percentages. The discrepancy rate was calculated as the number of discrepancies, divided by the number of medications listed in the SMR. To investigate the association of different factors for the rate of discrepancies, Quasi-Poisson regression models were fitted. Quasi-Poisson was used to account for underdispersion in the models (all dispersion estimates were between 0.16 and 0.30). Factors included in the models were sex, age (<65 years, 65–79 years, or ≥80 years), time since last update of the SMR (tertiles), source of last SMR update (hospital, outpatient clinic, or GP), time of admission (during or outside normal working hours), assistance with medication dispensing (yes or no), and triage level (level 1–2, level 3, or level 4). Models were fitted for each factor including the specific factor with age and sex to adjust for confounding. However, age and sex models were not adjusted if they only included their specific factor. Results from the models are presented as incidence rate ratios (IRR) with confidence intervals (CI). Additionally, models were repeated with excluding discrepancies due to order not in use in the rate calculation. Bonferroni correction was used to account for multiple testing by upscaling *p*-values with number of tests, all upscaled *p*-values larger than 1 are set to 1. Data were processed using Microsoft Excel XLSTAT. All calculations and statistical analyses were performed in R 3.6.1 [34]. For all statistical tests, *p* < 0.05 was considered statistically significant.

## 5. Conclusions

In a cohort of 100 patients consecutively admitted to the ED, we found that 81% of patients had ≥1 discrepancy between the SMR and patients’ actual use of medication. Age < 65, longer time since prior SMR update, and patient self-dispensing were associated with a higher frequency of discrepancies. During the study, 84% of the patients were available for medication reconciliation by a clinical pharmacist within normal working hours. The high frequency of discrepancies serves as a caution to clinicians who rely on the SMR when obtaining a medication history in daily practice. Future studies should utilize risk stratification models to identify patients with the highest risk of serious discrepancies leading to adverse clinical outcomes.

## Figures and Tables

**Table 1:** Patient characteristics of the included patients (*n* = 100).

Demographic Data	Median (IQR) or *n* (%)
Sex (men)	52 (52)
Age (years)	66.5 (53–80)
Admitted during normal working hours 8:00 a.m.–3:00 p.m.	48 (48)
Admitted outside normal working hours 3:01 p.m.–07:59 a.m.	52 (52)
Referred to the ED by a GP or Outpatient clinic	37 (37)
Referred to the ED by an emergency or out-of-hours service healthcare professional	55 (55)
Self-referral to the ED	8 (8)
Triage level ≥ 3	78 (78)
Length of hospital stay	2 (1–4)
Patients with a hospital interaction within 90 days before index admission	66 (66)
eGFR mL/min/1.73 m^2^	83 (56–90)
<60 mL/min/1.73 m^2^	29 (29)
Medication listed in the SMR	10 (7–13)
Medication used (regularly scheduled and PRN)	8 (5–11)
Medication used (regularly scheduled)	6 (3–9)
Patients using ≥1 regular medications	93 (93)
Patients using ≥5 regular medications	63 (63)
Days since the last SMR update *	59 (14–154)
<30 days since the last SMR update	35 (35)
<31–89 days since the last SMR update	16 (16)
≥90 days since the last SMR update	39 (39)
GP completed last update of the SMR *	24 (26)
Help with medication dispensing	29 (29)

* *n* = 92; ED, emergency department; eGFR, estimated glomerular filtration rate; GP, general practitioner; SMR, Shared Medication Record; PRN, Pro re nata.

**Table 2:** Types and number of discrepancies.

Types of Discrepancies	Discrepancies, *n* (%)	Patients, %
Order not in use	157 (65)	61
Incorrect dose frequency	37 (16)	24
Omission of order	29 (12)	15
Duplicate order	9 (4)	9
Incorrect dosage	8 (3)	6

**Table 3:** Factors associated with prescribing discrepancies between the Shared Medication Record (SMR) and patients actual use of medications.

Covariate (Number of Patients)	Incidence Rate Ratio	Confidence Interval	*p*-Value
Age, years			
<65 (44) 65–79 (30) ≥80 (26)	Ref 0.58 0.49	Ref 0.48–0.71 0.38–0.92	Ref <0.001 <0.001
Female Male 65–79 (52)	Ref 0.96	Ref 0.80–1.15	Ref 1.00
*All models are adjusted for age and sex*			
Days since the last SMR update *			
First tertile: 0–27 (33) Second tertile: 28–114 (28) Third tertile: ≥115 (29)	Ref 1.16 1.53	Ref 0.96–1.40 1.29–1.82	Ref 1.00 <0.001
Who updated the SMR last *			
Hospital (37) Outpatients clinic (29) GP (24)	Ref 1.02 1.19	Ref 0.84–1.23 0.98–1.43	Ref 1.00 0.836
Time of admission to the ED			
During normal working hours (48) Outside normal working hours (52)	Ref 1.04	Ref 0.87–1.24	Ref 1.00
Help with medication dispensing			
No (71) Yes (29)	Ref 0.31	Ref 0.24–0.39	Ref <0.001
Triage level			
1 or 2 (23) 3 (51) 4 (26)	Ref 0.95 1.16	Ref 0.75–1.19 0.90–1.49	Ref 1.00 1.005

* *n* = 90, ED, emergency department; GP, general practitioner; SMR, Shared Medication Record. Note: The *p*-values are adjusted for multiple comparisons.

**Table 4:** Time intervals for admission and/or discharge in relation to the clinical pharmacists’ normal working hours.

Time	Number of Patients	Patients with ≥1 Prescribing Discrepancy, *n* (%)
Admitted during normal working hours (8.00 a.m.–3.00 p.m.)	49	37 (76)
Admitted outside normal working hours (3.01 p.m.–7.59 a.m.), but still admitted the following morning (until at least 9.30 a.m.)	35	27 (77)
Admitted and discharged outside normal working hours (3.01 p.m.–7.59 a.m.)	16	15 (94)

## Data Availability

The data presented in this study are not publicly available due to Danish legislation.

## Appendix A

**Table A1:** Distribution of discrepancies categorized by Anatomic Therapeutic Index (ATC).

ATC-Drug Group (Level 2)	Description	Number of Discrepancies, n (%)
A02	Drugs for acid related disorders	25 (22.7)
N02	Analgesics	13 (11.8)
C09	Agents acting on the renin-angiotensin system	11 (10.0)
A06	Drugs for constipation	5 (4.5)
B01	Antithrombotic agents	5 (4.5)
A12	Mineral supplements	5 (4.5)
C10	Lipid modifying agents	5 (4.5)
C01	Cardiac therapy	5 (4.5)
R03	Drugs for obstructive airway diseases	4 (3.6)
B03	Antianemic preparations	4 (3.6)
J01	Antibacterials for systemic use	4 (3.6)
N03	Antiepileptics	4 (3.6)
N05	Psycholeptics	3 (2.7)
A11	Vitamins	3 (2.7)
N06	Psychoanaleptics	2 (1.8)
A10	Drugs used in diabetes	2 (1.8)
M01	Anti-inflammatory and antirheumatic products	2 (1.8)
H02	Corticosteroids for systemic use	2 (1.8)
R01	Nasal preparations	1 (0.9)
C03	Diuretics	1 (0.9)
M03	Muscle relaxants	1 (0.9)
S01	Ophthalmologicals	1 (0.9)
L01	Antineoplastic agents	1 (0.9)
D01	Antifungals for dermatological use	1 (0.9)

**Table A2:** Factors associated with prescribing discrepancies between actual use of medication compared to dispensed medication in the shared medication record (SMR).

Covariate (Number of Patients)	Incidence Rate Ratio	Confidence Interval	*p*-Value
Age, years			
<65 (44) 65–79 (years) (30) ≥80 (years) (26)	Ref 0.67 0.53	Ref 0.45–0.99 0.33–0.86	Ref 0.514 0.119
Female Male 65–79 (52)	Ref 0.98	Ref 0.68–1.43	Ref 1.00
*All models are adjusted for age and sex*			
Days since the last SMR update *			
First tertile: 0–27 (33) Second tertile: 28–114 (28) Third tertile: ≥115 (29)	Ref 1.27 1.14	Ref 0.85–1.89 0.76–1.73	Ref 1.00 1.00
Who updated the SMR last *			
Hospital (37) Outpatients clinic (29) GP (24)	Ref 1.03 1.22	Ref 0.69–1.54 0.82–1.81	Ref 1.00 1.00
Time of admission to the ED			
During normal working hours (48) Outside normal working hours (52)	Ref 2.59	Ref 1.84–3.63	Ref <0.001
Help with medication dispensing			
No (71) Yes (29)	Ref 0.18	Ref 0.10–0.33	Ref <0.001
Triage level			
1 or 2 (23) 3 (51) 4 (26)	Ref 0.97 0.79	Ref 0.62–1.53 0.45–1.37	Ref 1.00 1.00

* *n* = 90, ED, emergency department; GP, general practitioner; SMR, Shared Medication Record. Note: The *p*-values are adjusted for multiple comparisons.
